# Development and characterization of a unique anti‐IgE mouse monoclonal antibody cross‐reactive between human and canine IgE

**DOI:** 10.1002/iid3.531

**Published:** 2021-09-17

**Authors:** Akiko Kumagai, Takuya Nara, Mizuho Uematsu, Yoko Kakinuma, Takashi Saito, Kenichi Masuda

**Affiliations:** ^1^ Animal Allergy Clinical Laboratories Inc. Sagamihara Kanagawa Japan; ^2^ Vaccine Innovation Laboratory, RIKEN Cluster of Science, Technology and Innovation Hub, RIKEN Baton Zone Program, RIKEN Yokohama Institute Yokohama Kanagawa Japan

**Keywords:** animals, epitopes, humans, IgE | monoclonal antibody

## Abstract

**Background:**

The efficacy assessment of human anti‐IgE monoclonal antibodies (mAbs) in animal models before clinical trials is hampered due to the lack of cross‐reactivity of anti‐IgE mAbs between species.

**Objective:**

We developed CRE‐DR (an anti‐dog IgE monoclonal antibody), an anti‐IgE mouse mAb that recognizes canine and human IgE, and then examined its IgE specificity and cross‐reactivity between three animal and human species.

**Methods:**

After mouse immunization with a synthetic peptide derived from canine IgE (^282^NTNDWIEGETYYC^294^), we generated a hybridoma producing CRE‐DR. The CRE‐DR purified from the ascites of hybridoma‐inoculated mice was used for ELISA and Western blot analysis to examine reactivity to dog, human, and rodent IgEs as well as recombinant bovine serum albumin (BSA)‐conjugated to canine, human, and rodent IgE amino acid peptides corresponding to the immunizing sequence. We then performed enzyme‐linked immunosorbent assays (ELISAs) for dog IgE using sera from dogs with atopic dermatitis (AD) after inhibition with canine IgE and IgG. The amino acid sequence recognized by CRE‐DR was identified by ELISA using synthetic peptides.

**Results:**

CRE‐DR is a monoclonal mouse IgG1κ specific for dog IgE, and the ELISA values in atopic dog sera were inhibited by dog IgE, but not dog IgG. The binding of CRE‐DR to human IgE was relatively maintained, but not to rodent IgEs, which results were confirmed with the BSA‐conjugated IgE peptides of the various species. The CRE‐DR reactivity was supported by the comparison of amino acid sequence of CRE‐DR epitope, DWIEGETYYC, in dog IgE; one, two, and three amino acids were substituted in the human, rat, and mouse IgE epitopes, respectively.

**Conclusions and Clinical Relevance:**

CRE‐DR is a mAb cross‐reactive to dog and human IgEs, which can allow the use of a dog model of allergy to test the efficacy of a CRE‐DR‐derived anti‐IgE therapeutic mAb before human clinical trials.

## 1 INTRODUCTION

The discovery of IgE as a key molecule in type I hypersensitivity has had a great impact on our understanding of the pathogenesis of allergic diseases.^1^ IgE binds to the high‐affinity IgE receptor (FcεRI) expressed mainly on mast cells and basophils.^2^ When IgE molecules are cross‐bridged, mast cells degranulate and release multiple inflammatory mediators, such as histamine, and induce the acute inflammation typical of type I hypersensitivity.^3^ The targeting of IgE by anti‐IgE monoclonal antibodies (mAbs) has been successful to control clinical signs in IgE‐mediated allergic diseases.^4^, ^5^, ^6^ Omalizumab was the first approved anti‐IgE therapeutic mAb shown to reduce serum IgE levels in human patients with asthma^7^ and chronic spontaneous urticaria.^8^ Ligelizumab, another anti‐IgE mAb, has a higher effect on serum IgE reduction due to a higher affinity for IgE than omalizumab.^5^ However, as both antibodies seem unable to eliminate IgE‐producing B cells,^5^, ^9^ their effects to control clinical symptoms of IgE‐mediated disease appear limited. In contrast, quilizumab was a different anti‐IgE mAb that targeted IgE‐producing B cells by linking to the M1 prime protein, an IgE membrane‐binding protein on B cells, but it did not bind to serum IgE. Unfortunately, serum IgE was decreased in Phase II clinical trial of human patients receiving quilizumab,^10^ thus indicating that the quilizumab also bound to serum IgE before it reached IgE‐producing B cells, and this led to the cancellation of its development.

Animal models of allergy could be used to reduce the risk of failure before testing anti‐IgE therapeutic mAbs in human clinical trials. Mice transgenic for the human FcεRIα gene have been used to evaluate the inhibition of anti‐IgE humanized antibodies on recombinant human IgE bound to the neo‐expressed human FcεRIα *in vivo*.^5^ Unfortunately, such an artificial model that only expresses a single human molecule would be insufficient to investigate complex immune reactions *in vivo*, such as antibody‐dependent cellular cytotoxicity, antibody‐dependent cellular phagocytosis, or the effect of anti‐IgE therapeutic mAbs. In contrast, the dog has been long known to suffer from spontaneous IgE‐mediated reactions that result in clinical disease manifestations, such as atopic dermatitis (AD).^11^, ^12^ Serum antigen‐specific IgE increased after allergen exposure and AD flares in dogs.^12^ The IgE‐mediated origin of such reactions was proven by basophil‐histamine release.^11^ A comparison of immunologic characteristics of canine and mouse models of AD confirms the high translational value of canine AD for the human disease homologue.^13^ Thus, dogs with AD would be a valid spontaneous model of type I hypersensitivity to evaluate the complex immune effects of human anti‐IgE mAbs. However, for this purpose, human anti‐IgE mAbs would need to be cross‐reactive with canine IgE.

Since the amino acid sequence between IgEs of humans and animals is not highly homologous,^14^ it has been difficult to generate an anti‐IgE antibody cross‐reactive between canine and human IgE. Nevertheless, the IgE amino acid sequence that binds to the FcεRIα appears relatively well‐conserved among humans and animals,^15^ and a mAb targeting this sequence would be expected to recognize both canine and human IgE. The fine epitope of omalizumab, ^424^HLP^426^ of human IgE—the IgE binding region to FcεRIα—^6^ is also identical in dog IgE, but the reactivity of omalizumab to canine IgE has never been reported. Omalizumab also binds to conformational epitope,^5^ and due to the existing conformational changes of IgE,^16^ structural differences in IgE would likely have an influence on the binding of omalizumab to dog IgE. For these reasons, a therapeutic mAb cross‐reactive between canine and human IgE has not been characterized and developed to date.

In this study, we generated a unique anti‐IgE mAb, CRE‐DR (an anti‐dog IgE monoclonal antibody), cross‐reactive between dogs and humans, against a peptide well‐conserved between species. The reactivity of CRE‐DR was investigated against canine, human, and rodent IgE. Finally, the CRE‐DR epitope was mapped to confirm its cross‐reactivity.

## 2 METHODS

### 2.1 Development of the CRE‐DR, anti‐canine IgE mAb

The peptide for mouse immunization consisted of a 13‐mer peptide whose NTNDWIEGETYYC sequence corresponds to the position 282–294 segment of the dog IgE heavy chain (GenBank No. AAA56797.1), because the site was protruded outside in the IgE structure (Protein Data Bank[PDB], identification code 1F6A^17^) and suspected to have a possible antigenicity in a previous report to develop antiserum against a neighboring site.^18^ This synthetic peptide, conjugated with keyhole limpet hemocyanin (Sigma‐Aldrich Japan Inc.), was injected at the tail base of BALB/c mice. After fusing external iliac lymph node cells and SP2 mouse myeloma cells, a hybridoma producing a monoclonal mouse IgG reactive to both dog IgE (Bethyl Laboratories, Inc.) and rat IgE (Invitrogen) was selected by enzyme‐linked immunosorbent assay (ELISA) on the cell culture supernatant using donkey‐anti‐mouse IgG and goat‐anti‐mouse IgG light chain polyclonal antibodies (Jackson ImmunoResearch Laboratories, Inc.). The obtained mAb, named CRE‐DR, was purified with protein G sepharose (Cytiva) from the ascites of mice intraperitoneally inoculated with this hybridoma. The final concentration of purified CRE‐DR was measured by absorbance at 280 nm. The CRE‐DR mouse IgG subclass was determined using the MAb Isotyping Test Kit (Bio‐Rad Laboratories, Inc.). The experiments for hybridoma development and mouse ascites collection had been approved beforehand by the Animal Care and Use Committee of the Cell Engineering Corporation (approval number; S‐233) and T.K. Craft Ltd. (approval number; AE190213), respectively.

### 2.2 Reactivity of CRE‐DR to dog, human, rat, and mouse IgE in ELISA

Commercially available dog IgE (Bethyl Laboratories Inc.), human IgE (Merck KGaA), rat IgE (Thermo Fisher Scientific, Inc.), mouse IgE (BD Pharmingen), and dog IgG (Cappel) were used for fluorescence ELISAs with biotinylated CRE‐DR (EZ‐Link NHS‐PEG12‐Biotin) (Thermo Fisher Scientific), using the Gemini XPS microplate reader (Molecular Devices, LLC.) and SoftMax Pro software (Molecular Devices), as reported previously.^19^

### 2.3 Reactivity of CRE‐DR in Western blot analysis

SDS‐PAGE was performed with a polyacrylamide linear gradient gel (5%–20%) for IgE reagents of dog (Bethyl Laboratories), human (Merk KGaA), mouse (BD Pharmingen), dog IgG (Cappel), and canine serum albumin (MP Biomedicals) at 2 μg/lane for Coomassie Brilliant Blue staining and 0.1 μg/lane for Western blot analysis under both reduced and nonreduced conditions. For Western blot analysis, the gel was transferred to a PVDF membrane (Bio‐Rad Laboratories) and reacted with biotinylated CRE‐DR at 1 μg/ml in 10‐fold diluted blocking buffer (KAC Co., Ltd) after blocking. The membrane was washed with TBS‐0.05% Tween 20 (TBS‐T), and bands were visualized with streptavidin‐conjugated alkaline phosphatase (F. Hoffmann‐La Roche, Ltd.) and 1‐Step™ NBT/BCIP Substrate Solution (Thermo Fisher Scientific).

### 2.4 Reactivity of CRE‐DR to partial peptides of dog, human, and mouse IgE

We produced a recombinant protein with a molecular weight (MW) of 71 kDa by expression in *Escherichia coli* BL21star (DE3) transfected with a pET‐15b vector carrying an N‐terminal His‐tag sequence followed by a thrombin site and the cloning sites of NdeI and XhoI, between which a synthesized DNA was inserted. From the translated amino‐ (N‐terminus) to the carboxy‐terminus (C‐terminus), this synthesized DNA encoded a segment of bovine serum albumin (BSA) (GenBank No. BC142272.1; positions 108 to 1856), a GS‐linker (GGSGGTGGSGS), and the dog IgE peptide used in the mouse immunization. After the His‐tagged recombinant protein was purified by nickel resin, the final concentration in PBS was determined by the Pierce BCA Protein Assay Kit (Thermo Fisher Scientific). Similarly, we synthesized DNAs for the ^433^GTRDWIEGETYQC^445^ (GenBank No. AAB59424) and ^411^VAKDWIEGYGYQC^423^ (GenBank No. BAQ55489.1) peptides, which correspond to the ^282^NTNDWIEGETYYC^294^ immunizing dog IgE sequence in the human and mouse IgE, respectively, used as recombinant human and mouse proteins (i.e., BSA‐human and BSA‐mouse peptides). All of these procedures were outsourced to the GenScript Biotech Corporation. The cross‐reactivity of CRE‐DR to the peptides and to recombinant BSA (Pierce Chemical) was examined by ELISA.

### 2.5 Der f 2 inhibition ELISA

Recombinant silkworm‐expressed Der f 2 was prepared as previously reported (a kind gift of Zenoaq Nippon Zenyaku Kogyo, Co., Ltd.).^20^ We selected serum samples from four allergy‐suspected dogs (Dogs 1–4), which we had previously found to have high levels of Der f 2‐IgE (data available at 10.6084/m9.figshare.14229335) by ELISA with a slight modification of the previous method using a polyclonal goat anti‐dog IgE antiserum (Bethyl Laboratories).^21^ Der f 2‐coated plates and the preincubated canine sera at 56°C for 15 min were used to determine serum levels of Der f 2‐IgE by the ELISA using CRE‐DR, as described above. The reactivity of CRE‐DR to Der f 2‐IgE was also confirmed by Western blot analysis (data not shown). Using a standard curve generated from the reactivity of the CRE‐DR to the BSA‐dog peptide captured by plate‐coated anti‐His‐tag mAb (clone, HIS.H8) (Thermo Fisher Scientific), the concentrations of Der f 2‐specific IgE in the dog sera were calculated from the number of molecules of dog IgE (196 kDa MW)^22^ equimolecular to those of the BSA‐dog peptide (71 kDa MW).

Inhibition ELISAs were performed with dog IgE (Bethyl Laboratories) and dog IgG (Cappel) as inhibitors.^19^ The biotinylated CRE‐DR was incubated with dog IgE or dog IgG at different molar ratio, and the canine sera were tested using the Der f 2‐IgE ELISA described above. After setting the uninhibited Der f −2 IgE levels at 100%, the inhibition percentages of Der f 2‐IgE concentrations were then calculated.

### 2.6 Mapping of the CRE‐DR epitopes

The original peptide used for mouse immunization, and seven N‐ or C‐truncated fragments of this peptide, were synthesized by a manufacturer (Cosmo Bio Co., Ltd.). Each peptide was conjugated to a 6x His‐tagged GGSGGS‐peptide at its N‐terminus and a GGSGGS linker. To design a capture ELISA, the anti‐His‐tag mAb (clone, HIS.H8) was immobilized onto the plates to capture the peptides that were then detected by the biotinylated CRE‐DR, as described above.

## 3 RESULTS

### 3.1 CRE‐DR specificity to canine IgE

**Figure 1:**
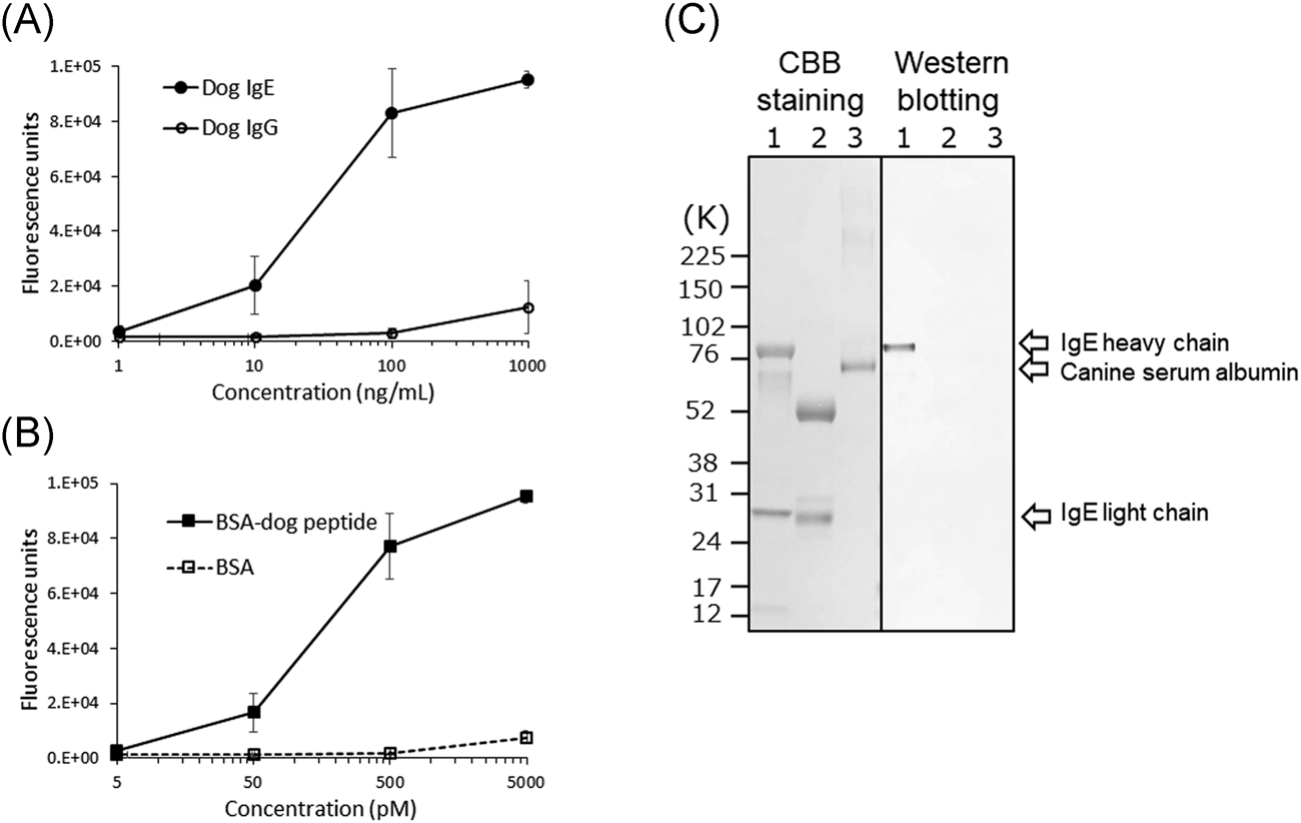
The reactivity of CRE‐DR to dog IgE were examined with enzyme‐linked immunosorbent assays (ELISAs) and Western blot analysis. (A) ELISAs with dog IgE‐ (●) or dog IgG‐coated (〇) plates showed that the CRE‐DR was reactive to dog IgE, but not to dog IgG. (B) CRE‐DR recognized recombinant bovine serum albumin (BSA) conjugated with the dog IgE immunizing peptide (BSA‐dog peptide) (■), but not recombinant BSA alone (**□**). These assays were independently repeated three times; the values represent the means, and the error bars are the standard deviation. (C) Western blot analysis for dog IgE (lane 1), dog IgG (lane 2), and canine serum albumin (lane 3) showed the specific reaction of CRE‐DR to dog IgE, especially heavy chain, not dog IgG. CBB, Coomasie Brilliant Blue.

Only one hybridoma cell line produced a mAb reactive to both dog and rat IgE. The mouse IgG subclass of CRE‐DR was IgG1κ. CRE‐DR specifically recognized dog IgE, but not dog IgG (Figure 1A,C), confirming its specificity to dog IgE.

The CRE‐DR binding to the BSA‐dog peptide increased in a dose‐dependent manner, while that to BSA alone remained low (Figure 1B). The maximum reactivity with the lowest background was obtained at 500 pM, which was determined to correspond to 35.5 ng/ml, after conversion using the MW of BSA‐dog peptide. These results support that CRE‐DR is a canine‐IgE‐specific mAb.

In the data of Western blot analysis (Figure 1C), CRE‐DR was only reactive to the heavy chain of dog IgE, which contained the target peptide of CRE‐DR, indicating specific reaction of CRE‐DR.

### 3.2 Allergen‐specific IgE detection in canine sera using CRE‐DR

**Figure 2:**
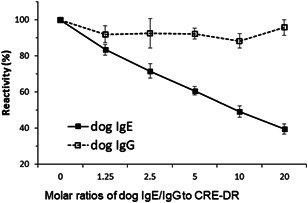
Inhibition percentages of dog IgE against Der f 2, one of major allergens of *Dermatophagoides farinae* for dogs, using inhibitors of dog IgE (■) and dog IgG (□) in enzyme‐linked immunosorbent assays (ELISAs). The inhibition percentages with dog IgE or dog IgG were calculated as a percentage of 100% without inhibitors. The experiments were repeated three times independently, and the means and standard deviations are shown.

Der f 2‐specific IgE in the four canine sera was quantitatively measured after conversion of the fluorescence units (FU)s to IgE concentrations in nanograms per milliliter, using a standard curve, as in Figure 1B. The four dogs were all found to have elevated Der f 2‐specific IgE levels (range: 991–1722 ng/ml). The preincubation of CRE‐DR with dog IgE inhibited the detection of Der f 2‐IgE in a dose‐dependent manner of IgE to between 35% and 42% of those without inhibitor. That with dog IgG did not (Figure 2), confirming the specificity of CRE‐DR for native dog IgE, even in sera that contain an amount of IgG larger than that of IgE.

### 3.3 Cross‐reactivities of CRE‐DR to human and rodent IgE

**Figure 3:**
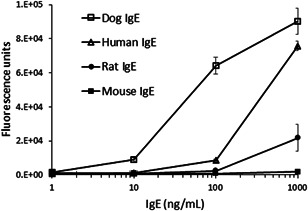
CRE‐DR reactivity to human and animal's IgE in enzyme‐linked immunosorbent assays (ELISAs) with immobilized IgE. Dog IgE was the most reactive to CRE‐DR in an IgE concentration‐dependent manner. Human and rat IgE were also recognized by CRE‐DR, but only at a high concentration of IgE and with a reduction of the reactivity. CRE‐DR did not bind to mouse IgE at any of the concentrations tested. The assay was independently repeated three times; the means and standard deviations are represented.

**Figure 4:**
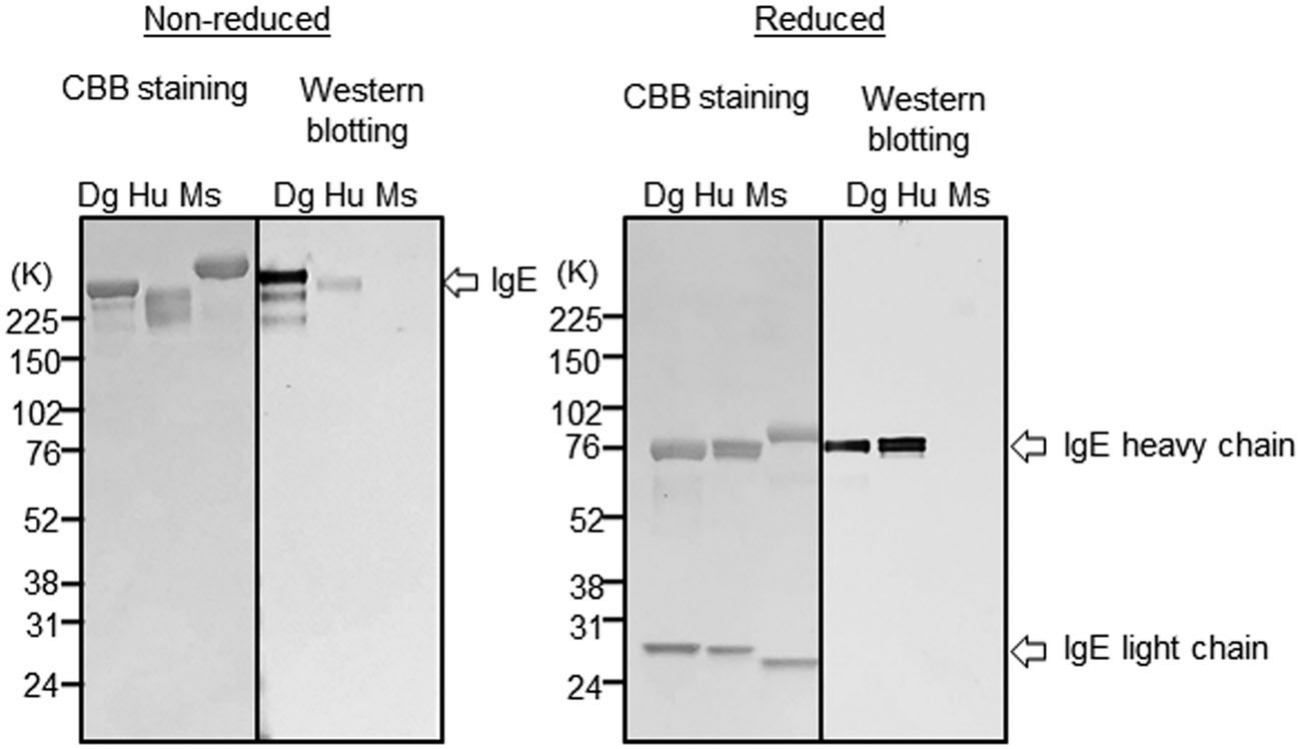
CRE‐DR reactivity to human and animal's IgE in Western blot analysis. Dog IgE was well‐reactive, showing the apparent band for IgE. In nonreduced condition, CRE‐DR reactivity to human IgE was reduced, which was recovered against the heavy chain of IgE in reduced condition, indicating the specificity of CRE‐DR binding to the target region of IgE. The reactivity of CRE‐DR to mouse IgE was not recognized. The assay was independently repeated two times and the representative data was shown. CBB; Coomasie Brilliant Blue; Dg, dog IgE; Hu, human IgE; Ms, mouse IgE

ELISA reactivity of CRE‐DR to human IgE was reduced only to lesser extent than that to canine IgE, but the reactivity to rat IgE was largely lost, and that to mouse IgE was completely disappeared (Figure 3). The tendency was confirmed by the results of Western blot analysis that showed an apparent band for dog IgE and a faint band for human IgE, but not for mouse IgE in the nonreduced condition, although in the reduced condition, the heavy chain of dog IgE and human IgE showed an apparent band with an equal density (Figure 4), indicating the specificity of CRE‐DR binding for the target region existing in IgE heavy chain.

**Figure 5:**
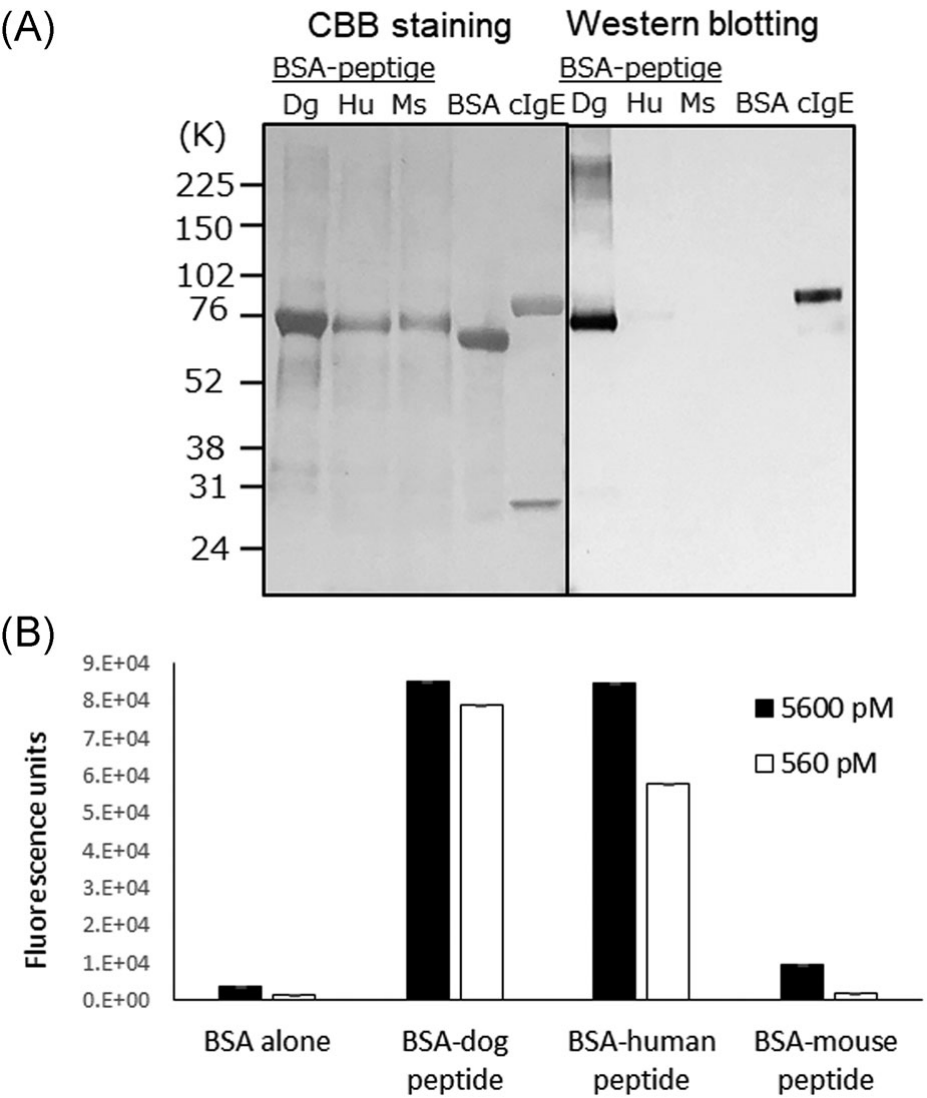
CRE‐DR reactivities to each His‐tagged peptide containing the epitope of dog, human, and mouse IgE, respectively, were visualized with Western blot analysis (A) and measured in capture enzyme‐linked immunosorbent assays using an antihistidine monoclonal antibody (B). (A) Western blot analysis showed an apparent band for bovine serum albumin (BSA)‐dog peptide (Dg) and a faint band for BSA‐human peptide (Hu), but not for BSA‐mouse peptide (Ms) and BSA. The data were represented in two independent experiments. (B) CRE‐DR recognized the BSA‐dog and BSA‐human peptides, but the reactivity to BSA‐mouse peptide was markedly reduced. The means are shown and the error bars indicate the standard deviation of three independent experiments.

The cross‐reactivity of CRE‐DR to human IgE was also confirmed with Western blot analysis using BSA‐peptides (Figure 5A), showing the reactivity of CRE‐DR to BSA‐dog peptide and BSA‐human peptide, not to BSA‐mouse peptide. The reactivities of CRE‐DR to the BSA‐dog and BSA‐human peptides in ELISA were nearly identical at the molarity of 5600 pM, while that to the BSA‐mouse peptide was approximately 10‐times lower (Figure 5B). At the concentration of 560 pM, the reactivities to BSA‐human and BSA‐mouse peptide were reduced (Figure 5B)—a trend similar to the difference in reactivities of the CRE‐DR to the dog, human, and mouse IgE at 1000 ng/ml (Figure 4). The tendency of CRE‐DR to cross‐react with BSA‐human peptide and not with BSA‐mouse peptide was also confirmed by Western blot analysis (Figure 5A).

### 3.4 Epitope mapping of CRE‐DR and reactivities to IgEs

**Figure 6:**
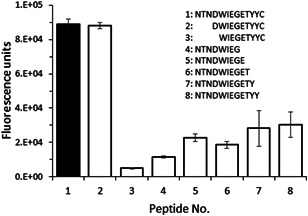
Epitope mapping of CRE‐DR using synthesized peptides after deletion of N‐ or C‐terminal amino acid(s) in enzyme‐linked immunosorbent assays (ELISAs). The fluorescence units of ELISAs against the peptides were compared to that of the original dog IgE peptide used for mouse immunization during the CRE‐DR development (Peptide No. 1). Peptide No. 2 was the only one with a reactivity similar to that of Peptide No. 1; its amino acid sequence is thus considered to represent the epitope of CRE‐DR. The error bars indicate the standard deviation of four independent experiments.

The CRE‐DR reactivity disappeared when four amino acids from the N‐terminus (Peptide No.3) and at least one amino acid from the C‐terminus (Peptide No. 4–8) were deleted from the original peptide (Peptide No. 1) (Figure 6). The reactivity to the Peptide No.2 (DWIEGETYYC) was almost identical to that of the original, confirming that it contains the main epitope of CRE‐DR.

One amino acid difference was found between the canine and human CRE‐DR epitopes: at position 293 in the dog IgE sequence, the tyrosine (Y) is replaced by a glutamine (Q) in the human one (GenBank No. AAB59424) (Table 1). There are two and three additional amino acid changes when comparing the canine to the rat and mouse epitope sequences, respectively.

**Table 1:** Comparison of amino acids of CRE‐DR epitope among IgEs of humans and animals

Species	Amino acid sequence	GenBank No.
Dog	D W I E G E T Y Y C	A A A 5 6 7 9 7.1
Human	D W I E G E T Y **Q** C	A A B 5 9 4 2 4
Rat	D W I E G E **G** Y **Q** C	A A A 4 1 3 6 4.1
Mouse	D W I E G **Y G** Y **Q** C	B A Q 5 5 4 8 9.1

*Note*: In bold and underlined are the amino acids that differ with those of the dog IgE.

## 4 DISCUSSION

The anti‐IgE mouse IgG1κ mAb CRE‐DR was specific for dog IgE—but not dog IgG—and cross‐reactive with human, but not rodent IgE, which were confirmed by ELISAs and Western blot analysis. The DWIEGETYYC amino acid sequence of the CRE‐DR epitope on dog IgE is well‐conserved with that of human IgE, as there is only one amino acid difference (Table 1). Indeed, human IgE and the BSA‐human peptide were still recognized by CRE‐DR, while the reactivity to mouse IgE and the BSA‐mouse peptide, with its three amino acid substitution in the CRE‐DR epitope, was almost completely lost. The CRE‐DR specificity for dog IgE was also confirmed with the inhibition ELISAs using dog serum from clinical cases of allergy, indicating that CRE‐DR could react not only with IgE reagents but also native dog IgE.

Since CRE‐DR was found to be reactive to dog and human IgEs, CRE‐DR cross‐reactivity to IgEs of the other animals could be speculated when compared the amino acid sequence of CRE‐DR epitope among those of animals’ IgEs. Pig IgE precursor (GenBank: AAC48776.1) showed the same amino acid sequence of CRE‐DR epitope as dog IgE, presumably recognized by CRE‐DR. Cow IgE constant region (GenBank: ANN46381.1) showed one amino acid substitution (Iso to Val at the position of 287) in the dog peptide. Cat IgE (GenBank: AAD20023.1) showed two amino acid substitutions (Iso to Val at 287 and Tyr to Gln at 292) in the dog peptide. The substitution of one amino acid will not be considered as a big obstacle for CRE‐DE cross‐reactivity, since Iso and Val are similar in physiological properties of the amino acids. An amino acid substitution in the dog peptide from Tyr to Lys at 293 was observed in horse IgE (GenBank: CAC44498.1), which physiological properties are not similar, affecting the binding of CRE‐DR. It is suspected hat CRE‐DR may lose the reactivity to the horse IgE in some degree.

The conformational structure of human IgE (PDB, 1F6A^17^) showed that the segment corresponding to the CRE‐DR epitope protrudes outward with the alpha helix (consisting of amino acids of DWIE, a N‐terminal part of CRE‐DR epitope) followed by the beta sheet (consisting of amino acics of TYQC, a C‐terminal part of CRE‐DR epitope) with a turn structure of glycine and glutamic acid (GE) between them. There is an N‐linked glycosylation site on an asparagine, 15 amino acid N‐terminally from the epitope. This N‐glycosylation, rich in mannoses, is indispensable for the “open” (oval shape of IgE constant region) structure of the CH3 domain that allows the binding of IgE to FcεRI.^23^ Therefore, the epitope site of the CRE‐DR could be extending outwardly, which might explain its immunogenicity. In fact, polyclonal antibodies have been generated against a peptide next to an epitope of CRE‐DR.^18^ The success of CRE‐DR development might be, at least in part, due to the selection of a region accessible to IgG antibodies.

There is some value in evaluating if the CRE‐DR, as a mAb whose epitope is clearly identified, would recognize the IgE of other mammalian species. Previously, we created the CRE‐DM, a rat mAb cross‐reactive between dog and mouse IgE,^19^ but its epitope was not determined. Since the anti‐human IgE omalizumab mAb recognizes three consecutive amino acids^6^ that are conserved between human and canine IgE, one would expect it to be cross‐reactive between human and dog IgE. In preliminary experiments, however, we found that the omalizumab did not bind to dog IgE, suggesting that any cross‐reactivity of anti‐IgE mAbs between humans and dogs requires the recognition of not only similar linear amino acid sequences, but also a similar conformation between those IgEs. In fact, it was reported that omalizumab recognized amino acids of human IgE in a nonsequential manner,^5^ which would affect its recognition of canine IgE. Since the need of conformational epitopes is a major obstacle to antibody development—at least with the existing technology—a mAb such as CRE‐DR cannot be easily generated.

Due to the unique ability of CRE‐DR to recognize both human and canine IgE, a dog model of IgE‐mediated allergy could be utilized to investigate the efficacy of anti‐IgE therapeutic mAbs derived from the CRE‐DR. A caninized CRE‐DR mAb could be first tested in dogs with spontaneous IgE‐mediated (i.e., “extrinsic”, increased serum IgE) AD to detect if this mAb could prevent or reduce clinical signs. Then, the results in dogs would be informative as preclinical data for the development of a humanized antibody derived from the CRE‐DR. Due to the closeness of IgE‐mediated allergic reaction between canine and human AD,^13^ a therapeutic success in dogs would reduce the inherent risk that exists when translating mouse studies to clinical trials in humans. This advantage has never been proposed by the other anti‐IgE antibody drugs such as omalizumab and ligelizumab, which cross‐reactivity to animal's IgEs was not shown.

In summary, we generated CRE‐DR, a unique mAb specific for dog IgE that also recognizes human IgE; such cross‐reactivity was confirmed by the recognition of both IgE and its derived recombinant peptides in dogs, humans, and mice, and the identification of the CRE‐DR epitope. An anti‐human IgE therapeutic mAb derived from the CRE‐DR could be developed after validation of the efficacy of a caninized CRE‐DR mAb on a canine model of IgE‐mediated AD. The caninized CRE‐DR itself will also be the first antibody drug for IgE in dogs. Thus, the cross‐reactivity of CRE‐DR should be highly considered to be useful for the treatment of allergy in both human and animals

## CONFLICT OF INTERESTS

K. Masuda is a stockholder and Chief Executive Officer of the Animal Allergy Clinical Laboratories, Inc.; K. Masuda and T. Saito are inventors in the patent application related to the CRE‐DR.

## ETHICS STATEMENT

There is no human clinical study. All mouse‐based experiments had been approved beforehand by the Animal Care and Use Committee of the companies performing the contract research.

## AUTHOR CONTRIBUTIONS

*Performed experiments and analyzed data*: Akiko Kumagai. *Performed experiments*: Takuya Nara, Mizuho Uematsu, and Yoko Kakinuma. *Analyzed and interpreted data, contributed the final writing*: Takashi Saito. *Conceived the original idea, planed and directed all the experiments, supervised the project, analyzed and interpreted data, wrote the manuscript*: Kenichi Masuda.

## Data Availability

The raw data are available at the repository (10.6084/m9. figshare.14229335).
